# Adipose Tissue-Derived Mesenchymal Stem Cells Increase Skin Allograft Survival and Inhibit Th-17 Immune Response

**DOI:** 10.1371/journal.pone.0076396

**Published:** 2013-10-04

**Authors:** Rafael Assumpção Larocca, Pedro Manoel Moraes-Vieira, Ênio José Bassi, Patrícia Semedo, Danilo Candido de Almeida, Marina Burgos da Silva, Thomas Thornley, Alvaro Pacheco-Silva, Niels Olsen Saraiva Câmara

**Affiliations:** 1 Laboratory of Transplantation Immunobiology, Department of Immunology, Institute for Biomedical Sciences, University of São Paulo, São Paulo, Brazil; 2 Laboratory of Clinical and Experimental Immunology, Division of Nephrology, Federal University of São Paulo, São Paulo, Brazil; 3 Instituto Israelita de Ensino e Pesquisa Albert Einstein Hospital, Renal Transplantation Division, São Paulo, Brazil; 4 Harvard Medical School, Department of Medicine, The Transplant Institute, Beth Israel Deaconess Medical Center, Boston, Massachusetts, United States of America; 5 Harvard Medical School, Department of Medicine, Division of Endocrinology, Beth Israel Deaconess Medical Center, Boston, Massachusetts, United States of America; Rutgers - New Jersey Medical School, United States of America

## Abstract

Adipose tissue-derived mesenchymal stem cells (ADSC) exhibit immunosuppressive capabilities both *in vitro* and *in vivo*. Their use for therapy in the transplant field is attractive as they could render the use of immunosuppressive drugs unnecessary. The aim of this study was to investigate the effect of ADSC therapy on prolonging skin allograft survival. Animals that were treated with a single injection of donor allogeneic ADSC one day after transplantation showed an increase in donor skin graft survival by approximately one week. This improvement was associated with preserved histological morphology, an expansion of CD4^+^ regulatory T cells (Treg) in draining lymph nodes, as well as heightened IL-10 expression and down-regulated IL-17 expression. *In vitro*, ADSC inhibit naïve CD4^+^ T cell proliferation and constrain Th-1 and Th-17 polarization. In summary, infusion of ADSC one day post-transplantation dramatically increases skin allograft survival by inhibiting the Th-17 pathogenic immune response and enhancing the protective Treg immune response. Finally, these data suggest that ADSC therapy will open new opportunities for promoting drug-free allograft survival in clinical transplantation.

## Introduction

Adipose tissue-derived stem cells (ADSC) are an attractive source of multipotent mesenchymal stem cells (MSC) for use in tissue engineering and clinical applications [1]. ADSC are characterized as a heterogeneous cell population expressing the surface markers CD73, CD44, CD90 and CD105, but not the hematopoietic lineage markers CD11c, CD31, CD34, CD45, CD80 and CD86. Essentially, their plasticity and ability to differentiate into mesenchymal origin tissues such as bone, cartilage and fat are considered the hallmark criteria for ADSC classification [2], [3]. Their relative abundance and easy accessibility within adult tissues make them ideal candidates for cell-based therapies. To this end, many authors have investigated their potential use to repair injured tissues [2]–[4].

In addition, several studies have shown that stem cells also posses immunomodulatory capabilities [5]–[10]. MSC inhibit T lymphocyte proliferation in mixed lymphocyte reactions (MLR) using third party stimulator cells polyclonal stimulation [11]–[13]. Furthermore, the administration of MSC was found to abolish graft-versus-host disease (GVHD) in human bone marrow transplantation, strongly suggesting that these cells can be used therapeutically *in vivo* [14].

The immunomodulation mediated by MSC requires prior activation of the MSC by immune cells through the release of proinflammatory cytokines such as IL-1α, IL-1β and IFN-γ [15], [16]. This prior activation has been shown to be important since IFN-γ receptor-1-deficient MSC are unable to exert any immunosuppressive effects [16]. However, once activated these MSC can release several soluble factors, such as indoleamine 2,3-deoxigenase (IDO), prostaglandin E_2_ (PGE_2_), inducible nitric-oxide synthase (iNOS) and IL-6 that have immunomodulatory effects on other cell types [12], [13], [17]. PGE_2_ was shown to have a negative effect on the maturation of dendritic cells, driving these cells instead to produce immunoregulatory IL-10 [18]. Similarly, IL-6 was reported to inhibit the maturation of dendritic cells through the down-regulation of costimulatory molecules such as CD40, and thereby block their ability to prime T cells [19], [20]. Moreover, MSC-derived IL-6 was shown to prolong neutrophil and lymphocyte survival [21], [22]. Overall, these data show the capacity of MSC to inhibit the immune response by restraining dendritic cell maturation and inducing a concomitant loss of function in NK, B and T cell compartments [23], [24].

More than that, MSC can induce the expansion of regulatory CD4^+^ T cells in the periphery [25], [26] while also inhibiting Th-17 cell generation [27], [28]. Accordingly, the use of ADSC has been widespread since they can be harvested easily via liposuction and then expanded *in vitro*. These ADSC can suppress T cell immune responses *in vivo* in the GVHD model [29] as well as in experimental models of autoimmune diseases [25], [30], [31]. However, the use of ADSC to induce transplant tolerance remains untested. Herein, we sought to study the therapeutic potential of allogeneic ADSC in orchestrating immunoregulation and prolonging skin allograft survival in a mouse model.

## Materials and Methods

### Animals

CBA/J (H-2^k^) and C57BL/6 (H-2^b^) mice were obtained from our Isogeneic Breeding Unit (Immunology Department, Institute for Biomedical Science, University of São Paulo – Brazil). All animals were used at 8–10 weeks of age. All protocols were conducted in adherence to the Brazilian Committee for Experimental Animals and were approved by the institutional ethics committee on animal use of the University of São Paulo (Protocol # 010, page 42 issue 2).

### Antibodies

Anti-CD3, anti-CD4 and anti-CD25 were purchased from BD Pharmingen; anti-CD11c, anti-CD31, anti-CD34, anti-CD40, anti-CD44, anti-CD45, anti-CD73, anti-CD80 and anti-CD86 were purchased from BioLegend; anti-Foxp3 was purchased from eBioscience.

### Skin Transplantation

Full-thickness skin grafts 1 to 2 cm in diameter were obtained from the tail-skin CBA/J donor mice and transplanted onto the back of C57BL/6 recipient mice [32]. Graft rejection was defined as the first day on which the entire graft was necrotic [32].

### Isolation and Characterization of ADSC

ADSC were collected from the epididymal fat of CBA/J mice and washed with phosphate-buffered saline (PBS). The fat was finely minced and digested with collagenase IV (Sigma) in a 37°C shaking water bath for 30 min. Then, the cell suspension was centrifuged and the cell pellet was resuspended in DMEM-low glucose (Invitrogen, EUA) supplemented with 10% fetal bovine serum (FBS, Gibco) and penicillin/streptomycin (Invitrogen). Cells were plated and incubated for 48 h at 37°C 5% CO_2_ and subsequently washed with PBS to remove residual no-adherent red blood cells. The adherent cells were maintained in culture for at least 4 passages prior to use. Immunophenotype characterization and multi-lineage differentiation potential were accessed in agreement with previous studies [33], [34].

### ADSC and Bone Marrow Mononuclear Cell Adoptive Transfers

On day +1 after skin transplantation, C57BL/6 mice were divided into three experimental groups: animals that received a single injection of 0.2 ml of PBS i.p. (Allo); animals that received 5×10^5^ CBA-ADSC i.p. (Allo-ADSC) or (B6-ADSC) or (Balb/c-ADSC); animals that received 5×10^5^ bone-marrow mononuclear cells i.p. (BMMC). In all experiments, we used 5 mice per group. The experiments were repeated three times.

### Cell Staining and Flow Cytometry

Cells obtained from the axillary lymph node were resuspended in FACS buffer (PBS with 2% FBS) and stained for flow cytometry analysis. To block Fc receptor-mediated binding of antibodies, mononuclear leukocytes were resuspended in FACS buffer with hamster anti-mouse CD16/32, clone 2.4G2 (Fc Block™, BD Bioscience) for 20 minutes. These cells were then washed, placed on ice for 30 minutes, and stained with fluorochrome-conjugated antibodies. Cells were washed twice in buffer and reserved for analysis. Intracellular staining for Foxp3 was performed on lymph node cells according to the manufacturer’s procedure (eBioscience). Intracellular staining for IFN-γ and IL-17 was performed on CD4^+^ T cells after activation with Leukocyte Activation Cocktail (BD Biosciences) and following cell permeabilization and fixation with the BD Cytofix/Cytoperm Fixation/Permeabilization Solution Kit (BD Biosciences) using the manufacturer’s protocol. For proliferation analyses, cells were stained with 5 µM CFSE using the manufacturer’s protocol. For cell acquisition, we used the BD FACSCanto II flow-cytometer (BD Biosciences). Data analysis for these experiments was performed using FlowJo software (Tree Star).

### Histology

The histological analysis of the skin graft was performed by staining 5 µm sections of paraffin embedded tissues with hematoxylin and eosin (HE) or Sirius red.

### Real-Time PCR

Skin and lymph node samples were initially snap-frozen in liquid nitrogen. Total RNA was isolated using the TRIzol Reagent (Invitrogen) according to manufacturer’s protocol. RNA concentrations were determined using NanoDrop (Thermo Scientific). First-strand cDNAs were synthesized using MML-V reverse transcriptase (Promega). Real-time PCR was performed using TaqMan PCR assays (Applied Biosystems) for the following genes of interest: IL-2 (Mm00434255_g1), IL-6 (Mm00446190_m1), IL-10 (Mm99999062_m1), IL-17 (Mm00439619_m1), Foxp3 (Mm00475156_m1), HPRT (Mm00446968_m1), IFN-γ (Mm00801778_m1) and TGF-β (Mm03024053_m1). Quantitative real-time PCR was performed via ABI PRISM 7300 Sequence Detection System (Applied Biosystems). Transcript levels were normalized to the expression of HPRT. Analyses were performed with the Sequence Detection Software 1.9 (SDS).

### MLR-based Suppression Assay

Stimulator mature DCs (5×10^4^ cells per well) from CBA/J mice were co-cultured with CFSE labeled naïve CD4^+^ T cells (1.5×10^5^ cells per well) isolated from draining lymph nodes of C57BL/6 mice using a CD4+ T cell positive selection kit (Miltenyi Biotechnology) according the manufacturer’s protocol. Cultures were performed with or without the addition of increasing concentrations of ADSC in a 96-well U-bottom plate. For the contact dependent assay, cells were incubated in a 96 transwell plate. Cells were incubated at 37°C for 96 hours. Cells were analyzed by flow cytometer. CD4^+^ T cell proliferation and DC activation marker expression were analyzed by FlowJo.

### Supernatant Cytokine Measurements by Bioplex®

A Bio-Plex mouse Plex cytokine assay kit (Bio-Rad laboratories) was used in conjunction with the Bio-Plex system array reader according to the manufacturer’s directions. The specific cytokines (IL-4, IL-10 and IFN-γ) were quantified. Standard curves for each of the analyzed cytokines were included in each run and sample concentrations were calculated using Bio-Plex Manager software version 4.0. Standard curves ranged from 32,000 to 1.95 pg/mL.

### Statistics

Data were analyzed using Prism5 (GraphPad Software Inc.), and the results were expressed as mean ± SEM. In the analysis, comparisons were made using the Mann Whitney *t test*. Survival curves were estimated by the Kaplan-Meier method and compared with a Log Rank test. P<0.05 was considered significant.

## Results

### Characterization of Adipose-derived Mesenchymal Stem Cells

**Figure 1:**
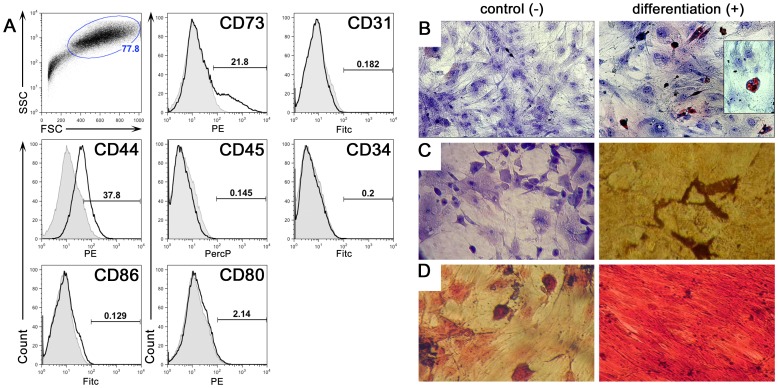
Isolation and *in vitro* characterization of ADSC. These cells were obtained after collagenase digestion (type V) of abdominal fat tissue from CBA/J mice and cultured in DMEM low-glucose supplemented with 10% FBS. Adherent cells were used in experiments through passage 8. Flow cytometry analyses from passage 6 are illustrated in panel (A). FSC by SSC indicates a homogeneous population in size and granularity. These cells were positive for CD44 and CD73 and negative for CD31, CD34, CD45, CD80 and CD86. More then 1×10^6^ events were acquired and the frequency of positive cells was determined using FlowJo. *In vitro*, these cells displayed the potential to differentiate into adipogenic, chondrogenic and osteogenic cells. ADSC were cultured in control (-) or differentiation (+) culture media, as described in the Methods. (B) Adipogenic differentiation: cells were stained with (Oil Red O) after 10 days under adipogenic culture condition and colored orange lipid vesicles could be observed (400×). (C) Osteogenic differentiation: cells were stained with (Von Kossa) after 28 days under osteogenic culture condition and colored calcium deposition is observed. (D) Chondrogenic differentiation: cells were stained with (Safranin O) after 21 days under chondrogenic conditions and intense red glycosaminoglycan staining was observed. Magnification 200×.

MSC can suppress immune responses both *in vitro* and *in vivo* [9], [25]. However, it is unknown whether this immunosuppressive capacity can be exploited for therapeutic advantage through the induction of tolerance to transplanted allografts. We therefore tested the hypothesis that MSC can generate a tolerogenic microenvironment imparting prolonged skin allograft survival. To address this hypothesis, we derived *in vitro* MSC from epididymal adipose tissue, which exhibit greater immunosuppressive capacity as compared to MSCs from bone marrow [35]. After three passages, the ADSC were collected and characterized by flow cytometry for CD11c, CD31, CD34, CD44, CD45, CD73, CD80 and CD86. As expected, the cells expressed CD44 and CD73 but not other markers (Figure 1A). Moreover, these ADSC exhibited characteristics of pluripotency as evidenced by their differentiation into adipocytes, chondrocytes as well as osteocytes under different culture conditions (Figure 1B).

### ADSC Administration Prolongs Allogeneic Skin Graft Survival

**Figure 2:**
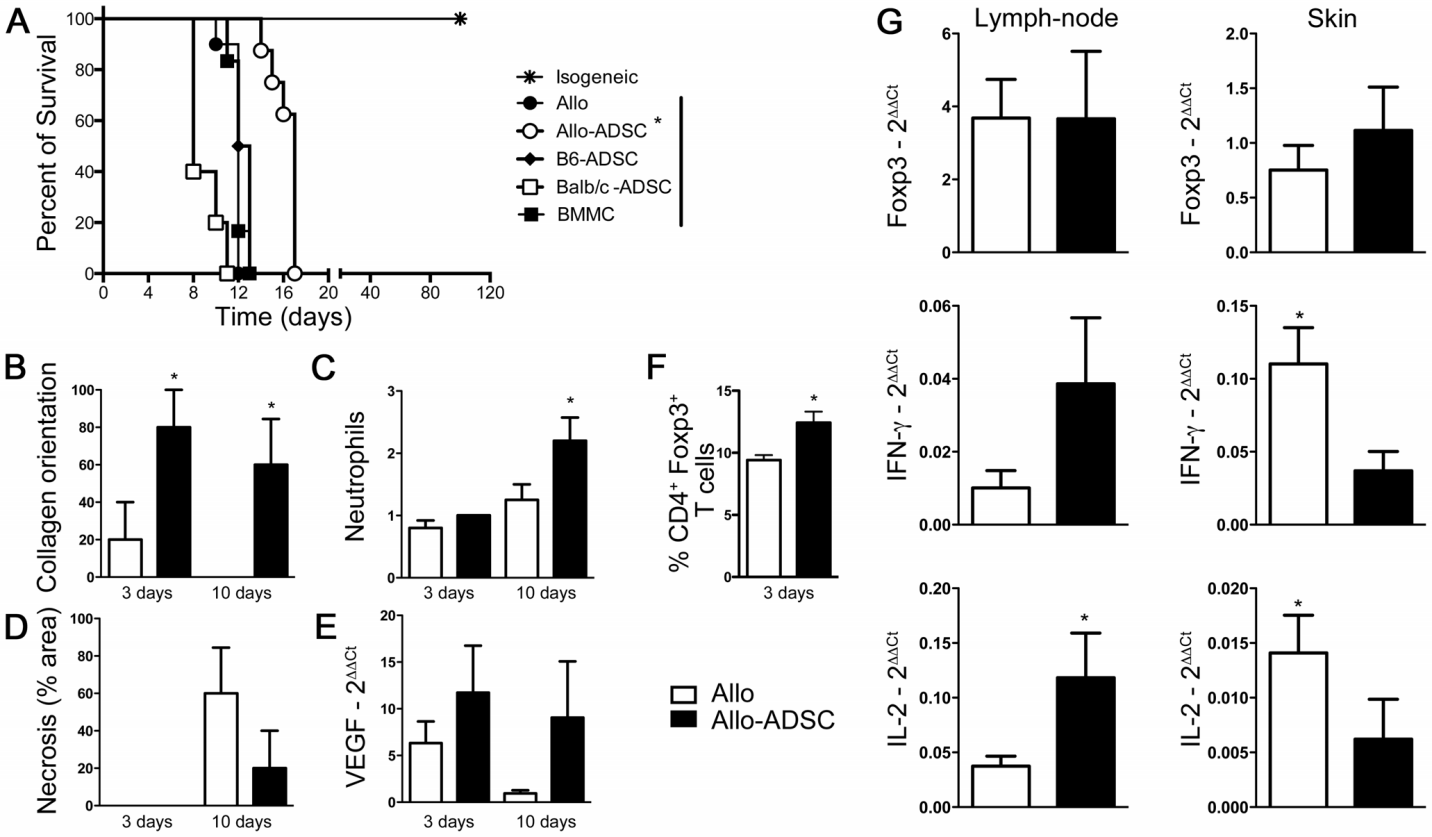
ADSC confer increased graft survival upon adoptive transfer. Allogeneic donor CBA/J skin allografts were transplanted onto C57BL/6 recipients. On day +1 following surgery, recipients were separated into three experimental groups: (A) (Allo-ADSC) were injected intraperitoneally with 5×10^5^ ADSC (n = 8); (BMMC) were injected intraperitoneally with 5×10^5^ bone marrow mononuclear cells (n = 6); (B6-ADSC) were injected intraperitoneally with 5×10^5^ ADSC from syngeneic C57BL/6 donors; (Balb/c-ADSC) were injected intraperitoneally with 5×10^5^ ADSC from a third party strain; (Allo) were intraperitoneally with PBS (n = 10). Isogenic skin transplants were used as controls (n = 5). Morphometric analyses from skin histology at day 3 and 10 show: (B) collagen orientation (Sirius red), (C) neutrophil infiltration and (D) necrosis. (E) VEGF quantification was performed by RT-PCR. (F) Numbers represent the percentage of Foxp3^+^ cells within gated CD4^+^ T cells on day 3 from axillary draining lymph nodes. (G) RT-PCR data showing Foxp3, IFN-γ and IL-2 expression in axillary draining lymph nodes and skin on the day of rejection. Data are represented as mean ± SEM; *n* = 3 independent experiments. *P<0.05.

To address the question of whether ADSC can prolong allogeneic skin graft survival, wild type C57BL/6 mice were transplanted with fully MHC-mismatch tail skin from CBA/J mice followed by the passive transfer of ADSC derived from donor-matched CBA/J mice (allo-ADSC), host-matched C57Bl/6 (B6-ADSC), or third-party Balb/c (Balb/c-ADSC). In the untreated control group that received a single intraperitoneal injection of PBS one day after transplantation, the median survival time (MST) of transplanted skin grafts was 12 days. Strikingly, a single injection of 5×10^5^ donor-matched allo-ADSC one day after transplantation prolonged skin graft survival to an MST of 17 days. In contrast, the transfer of 5×10^5^ B6-ADSC or Balb/c-ADSC did not show improved graft survival with an MST of 12.5 and 11 days, respectively (Figure 2A). Moreover, transfer of donor-matched mononuclear cells from bone marrow, which has a low fraction of MSC, failed to increase graft survival as compared to the untreated control (MST = 12 days, Figure 2A).

Consistent with prolonged skin allograft survival, allo-ADSC treated animals presented a healthier skin morphology with maintenance of the collagen orientation (Figure 2B), higher neutrophil infiltration (Figure 2C), and lower necrosis (Figure 2D) when compared to untreated control mice 10 days post-transplantation. These histological improvements were accompanied by an elevated expression of vascular endothelial grow factor (VEGF) as assessed by quantitative PCR (qRT-PCR) (Figure 2E) and an increase in the frequency of CD4^+^Foxp3^+^ regulatory T cells as determined by flow cytometry (Figure 2F) in the draining lymph node 3 days post-transplantation.

### ADSC Treatment Inhibits IL-2 and IFN-γ Expression in the Graft at the Time of Rejection

Given that skin transplants survived longer in allo-ADSC treated mice, we hypothesized that ADSC treatment would lead to a decrease in the expression of T cell effector cytokines such as IL-2 and IFN-γ. Moreover, this treatment could increase in the expression of the immunoregulatory molecules such as Foxp3, and favor the expansion of CD4^+^Foxp3^+^ T cells. To test this hypothesis, we initially quantified the mRNA levels of IL-2, IFN-γ and Foxp3 in the transplanted skin graft and draining lymph nodes of allo-ADSC treated and control mice on the day of each individual animal rejection. Interestingly, we found higher expression of IL-2 and IFN-γ in the skin graft obtained from the untreated group as opposed to the allo-ADSC treated group (Figure 2G). This increase in IL-2 and IFN-γ expression in the skin graft was inverted in the draining lymph nodes. However, we did not observe any difference in Foxp3 expression between the two groups (Figure 2G).

### ADSC Treatment Increases the Expression of IL-6, IL-10, IFN-γ, and Foxp3 in the Lymph Node, but Reduces IL-17 Expression in the Skin Graft

**Figure 3:**
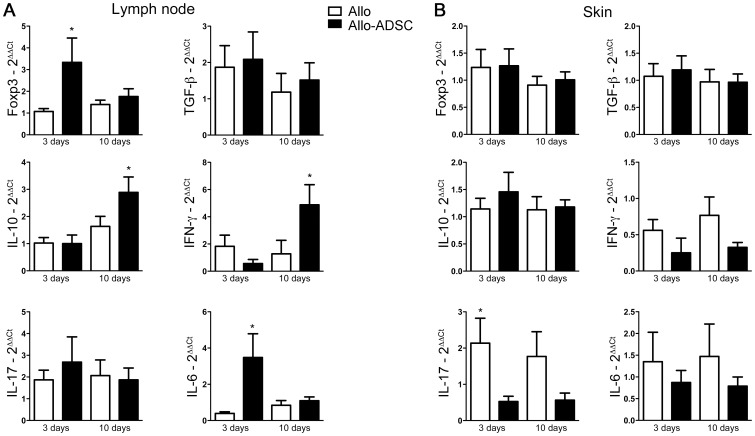
ADSC change the cytokine milieu *in vivo* and block Th-17 responses. C57BL/6 mice were grafted with full thickness allogeneic tail skin from CBA/J mice and treated or not with donor (CBA/J) ADSC. Tissues were analyzed on days 3 and 10 after transplantation. RNA was isolated from (A) draining axillary lymph nodes and (B) skin. Gene expression of Foxp3, TGF-β, IL-10, IFN-γ, IL-17 and IL-6 was assessed by quantitative RT-PCR. Samples were normalized by expression of an endogenous housekeeping gene (HPRT). Data are represented as mean ± SEM; *n* = 3 independent experiments done in triplicate, leading to a total of ≥10 independent values for each point. *P<0,05.

To closely examine the expression of molecules related to allograft tolerance and rejection, we harvested lymph nodes and skin grafts early (day 3) and preceding allograft rejection (day 10) post-transplantation. Interestingly, Foxp3 expression was higher in the draining lymph nodes of allo-ADSC treated mice than control mice on day 3 (Figure 3A), corroborating our earlier findings by flow cytometry (Figure 2F). We also observed higher IL-6 expression in the allo-ADSC treated group on day 3, which is not entirely unexpected due to ADSC’s known ability to secrete high levels of IL-6 [22] (Figure 3A). Interestingly we observed higher levels of IL-10 and IFN-γ in draining lymph nodes from allo-ADSC treated mice as compared to control mice on day 10 post-transplantation (Figure 3A). IFN-γ is often correlated as an effector cytokine produced by T cells during graft destruction, but it is also known that IFN-γ may have a suppressive function by inducing effector cell apoptosis [36]. Furthermore, it is also known that immunosuppression mediated by MSC depends on prior activation by proinflammatory cytokines [15], [16], [24]. ADSC can facilitate the production of IL-10 by other cells subtypes such as Treg, monocytes and dendritic cells [18], [37]. Regardless, untreated animals expressed higher levels of IL-17 in the skin graft on days 3 and 10 as compared to allo-ADSC treated animals (Figure 3B).

### ADSC Suppress Alloreactive Effector CD4^+^ T cell Proliferation and Differentiation *in vitro*

**Figure 4:**
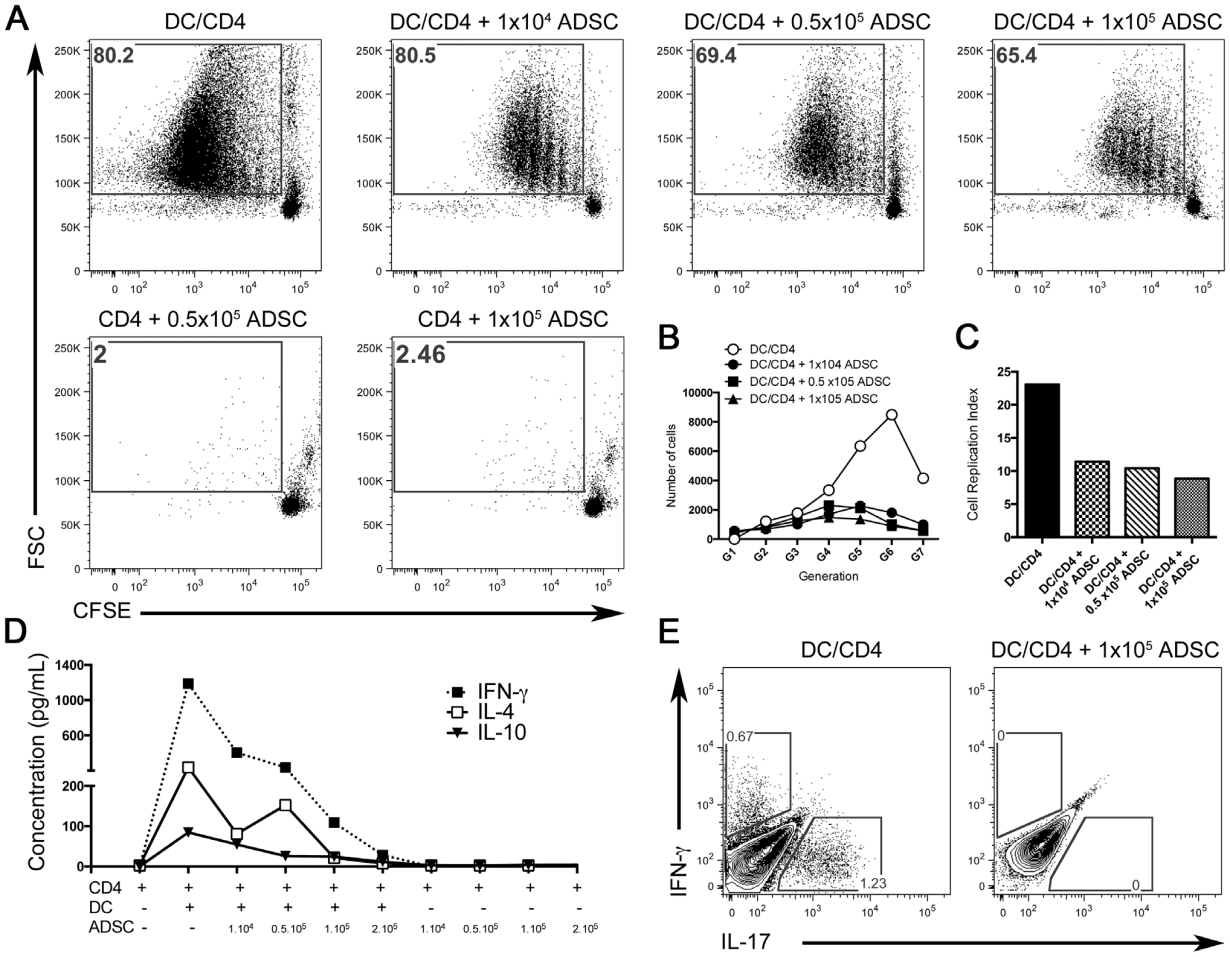
ADSC are effective suppressors of CD4^+^ T cell proliferation in an *in vitro* MLR co-culture and inhibit Th-1/Th-17 polarization. (A) In an MLR culture, naïve CD4^+^ T cells from the spleens of C57BL/6 mice were stimulated with mature dendritic cells from CBA/J mice for 4 days in the presence or absence of different concentration of ADSC. CD4^+^ T cell proliferation in these cultures was analyzed by flow cytometry. Gates represent the CFSE dilution peaks using FlowJo. (B) Expansion of cell generations was determined using FlowJo. (C) Cell proliferation relative index was determined using FlowJo. (D) Cytokine levels for IL4, IL-10 and IFN-γ from the culture supernatants were analyzed by Bioplex. (E) Intracellular staining for IL-17 and IFN-γ was performed on cultured T cells and analyzed by flow cytometry in gated CD4^+^ T cells using FlowJo.

To test the ability of the ADSC to suppress effector CD4^+^ T cells in a mixed leukocyte reaction (MLR), we performed an in vitro suppression assay. Splenic CD11c^+^ dendritic cells from CBA/J mice were co-cultured with purified C57BL/6 CD4^+^ T cells labeled with CFSE. After 5 days of culture, CD4^+^ T cells proliferated in response to allogeneic dendritic cells as measured by CFSE dilution (Figure 4A upper panel). The addition of increasing concentrations of CBA/J ADSC inhibited the proliferation of these CD4^+^ T cells in a dose-dependent manner (Figure 4A), reducing the frequency of proliferating responder cells by up to 20%. When we co-cultured CD4^+^ T cells with two different concentrations of ADSC alone, no proliferation was observed (Figure 4A lower panel). Even though the difference in the frequency of cells (represented by the gating) is not dramatic, we decided to evaluate the number of cells in each generation. Strikingly, when we analyzed the sixth and seventh generations, the number of cells that had proliferated in response to the allogeneic stimulus (DC+CD4) was 8-fold higher in comparison to the groups co-cultured with ADSC (Figure 4B). Moreover, when we measured the replication index, which determines the fold-expansion of only responding cells, we observed a more than 2-fold difference in proliferation (Figure 4C). Interestingly, this inhibition was not associated with a substantial increase in IL-10 production (Figure 4D). Additionally, this inhibition of proliferation was accompanied by a complete inhibition of effector T cell cytokine production, as demonstrated by an absolute abrogation of IFN-γ and IL-17 expression, the prototypic cytokines of Th-1 and Th-17 cells, respectively (Figure 4E).

### ADSC Suppress DC Costimulatory Molecules *in vitro*, in a Contact Dependent Manner

**Figure 5:**
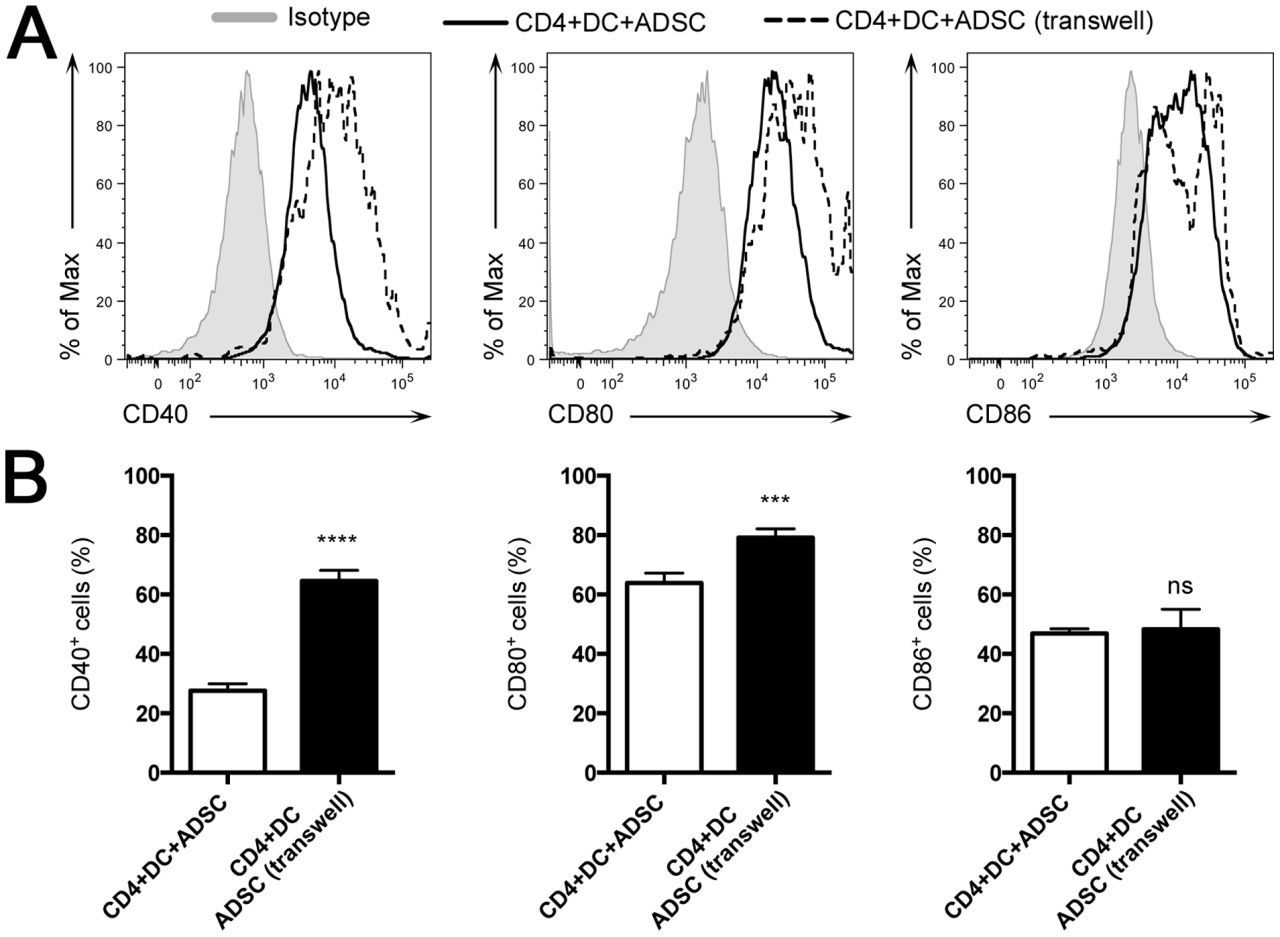
ADSC inhibit expression of costimulatory molecules by DC *in vitro*. (A) In an MLR culture, naïve CD4^+^ T cells from the spleens of C57BL/6 mice were co-cultured with mature dendritic cells from CBA/J mice in the presence of 1×10^5^ ADSC with contact or ADSC separated by a permeable membrane (transwell) for 4 days. Cells were analyzed by flow cytometry. (A) Histogram showing the expression of CD40, Cd80 and CD86 [Isotype control-tinted gray; CD4+DC+ADSC black line and CD4+DC+ADSC (Transwell)]. Data were analyzed using FlowJo.

To test whether the suppression that ADSC exerted on the CD4^+^ T cell proliferation was due to an indirect effect that ADSC had on DC, and to determine whether this effect was due to soluble factors or was contact dependent, we performed an MLR using a transwell system. We observed that CD11c^+^ cells cultured with contact had lower levels of costimulatory molecules, such as CD40 and CD80, as compared with CD11c^+^ cells cultured that were separated from ADSC by a cytokine-permeable transwell membrane (Figure 5A and B). We did not observe any difference in CD86 expression (Figure 5A).

## Discussion

Transplantation remains the best treatment option to correct certain types of organ failure and tissue damage (i.e. kidney, heart, lung, etc.). However, the long-term use of globally immunosuppressive drugs to prevent rejection carries with it some serious risks, including infection and cancer due to their lack of target specificity, as well as graft loss due to their toxicity [38]. Thus, identifying safe methodologies to induce donor-specific allograft survival is a top priority. One of the most attractive targets for such therapy is Treg, which have emerged as pivotal immunoregulators in the establishment of allograft tolerance [39]–[41]. While Treg improve graft survival in several experimental models, their low frequency under homeostatic conditions remains a roadblock to their therapeutic use. Consequently, finding strategies to expand Treg is essential for clinical success.

Herein, we exploited the immunoregulatory nature of ADSC to expand Treg and prolong skin graft survival. Similar to bone marrow-derived mesenchymal stem cells, ADSC found in adipose tissue possess extensive proliferative capacity and the ability to differentiate into multiple cell lineages [42], [43]. Due to the relative abundance of abdominal fat in normal individuals and the ability to isolate MSC after liposuction, we chose to use abdominal fat as the mesenchymal stem cell source [42]. Data from the literature shows that ADSC are better than bone marrow derived MSC and posses more potent immunomodulatory effects on DC [44], [45].

Strikingly, when allo-ADSC were administered, they significantly increased allograft survival compared with the untreated animals, although this did not result in permanent allograft tolerance. Moreover, transfer of ADSC from either a syngeneic donor or a third party donor (Balb/c) did not improve allograft survival, demonstrating that the prolongation of allograft survival is donor antigen specific. This is similar to results from previous studies showing that a donor specific transfusion (DST) into recipients can prolong allograft survival in both humans [46] and mice. Prolongation is enhanced when accompanied by anti-CD40L treatment [47].

Our data illustrate the potential of ADSC to suppress immune responses across a full MHC barrier. Furthermore, these data are in contrast to the results we obtained through the injection of mononuclear bone marrow cells, which we observed as having no beneficial effect on allograft survival as compared to the untreated controls. We presume that the low frequency of mesenchymal stem cells in the bone marrow makes it necessary to first expand or purify these cells *in vitro* prior to injection.

The fact that we do not see a long-term prolongation of the skin graft survival with ADSC might be due to ADSC rejection. Moreover, a recent study suggests that allogeneic MSC can be recognized by the innate and adaptive immune systems [48]. However, more detailed studies will be needed to clarify the precise mechanisms involved. We also believe that the immunogenicity of skin is an obstacle to achieving tolerance. Numerous models have demonstrated that it is difficult to generate permanent allograft tolerance to skin transplants [49]. In fact, it has been noted during whole hand transplantation that the skin was the first tissue to be rejected [50], [51].

Consistent with improved allograft survival, we observed a better histological morphology and preservation of collagen orientation with ADSC as compared to control treatment. Additionally, neutrophil infiltration was higher in ADSC treated mice as compared to the untreated group. While there are studies showing that neutrophils might contribute to allograft rejection [52]–[54], Larocca et al., have also shown the importance of neutrophil infiltration for graft acceptance in a skin transplant model [55]. Neutrophils produce vascular-endothelial grow factor (VEGF), an important growth factor needed for neovascularization and tissue repair [56]. Consistent with this, we observed increased VEGF expression in ADSC treated animals.

Additionally, we observed that the number of Treg in the draining lymph nodes from ADSC treated animals 72 hours after transplantation was increased as compared to untreated animals. We believe that this effect could be through direct expansion of the Treg pool, as it was already shown by our group that Treg proliferate *in vitro* in the presence of ADSC [25]. However, this profile was not maintained past day 10, when the number was equivalent between both groups. The hypothesis that these cells migrated to the graft was disproven when we failed to observe any difference in Foxp3 expression in the graft.

Next, we observed that the expression of IL-2 and IFN-γ transcripts was higher in the draining lymph node of the ADSC treated group, but lower in the graft of the untreated group. We believe that the ADSC migrated to the draining lymph nodes as our group has shown that ADSC injected into non-obese diabetic (NOD) mice migrate to the pancreatic lymph node (PLN), thus preventing insulitis and new onset diabetes [25]. Moreover, while IL-2 is an important growth factor for effector T cells, it is also an important growth factor for Treg. *In vitro* IL-2 and IFN-γ has been shown to be important in triggering MSCs to induce tolerance [57]. And, while it is well established that IFN-γ has an important role during the alloimmune response against the graft [58], it is also known that it can generate skin graft tolerance by activation of STAT-1 in a Treg population that is dependent on IFN-γ [59]. Furthermore, MSC-mediated immunosuppression is dependent on IFN-γ since MSC cells from IFN-γ receptor-1 knockout mice lack immunosuppressive capacity *in vitro* [16]. Moreover, skin allograft acceptance has been shown to be dependent on the presence of IFN-γ, as the addition of an anti-IFN-γ mAb or use of an IFN-γ knockout mouse was associated with prompt graft rejection [32].

We also observed that the expression of IL-6 was elevated in the ADSC treated group as compared to the untreated control group. While IL-6 has been shown to inhibit Treg, MSC-derived IL-6 also inhibits DC maturation, decreasing their capacity to prime T cells. IL-6 also delays apoptosis in neutrophils, providing one possible explanation for why we see an increase in neutrophil infiltration [19], [21], [22]. Thus, we hypothesize that these immature dendritic cells could be a source of the IL-10 observed in our studies.

Jointly with TGF-β, IL-6 drives the CD4^+^ T cell response to the Th-17 cell phenotype [60]. However, in these studies, we didn’t observe a difference in TGF-β expression between ADSC treated and control groups. However, we did observe a decrease in IL-17 expression in the graft of ADSC treated mice, as compared to untreated mice, 72 hours after transplantation, a difference that persisted through day 10. IL-17 producing Th-17 cells play an important role during inflammatory and pathogenic immune responses [61]. IL-17 can also induce early neutrophil apoptosis [20], offering a second possible explanation for our finding that untreated mice have decreased neutrophil infiltration.

Finally, we observed that ADSC suppressed CD4^+^ T cell proliferation and differentiation *in vitro* as well as expression of costimulatory molecules by DC (mainly CD40), a process that was contact dependent. The fact that we still observed some T cell proliferation in the presence of ADSC, but complete abrogation of cytokine production, is consistent with a previous publication showing that, in the absence of CD40 signaling, CD4^+^ T cells retain the capacity to proliferate but are unable to develop effector characteristics [62]. Thus, ADSC decreased the number of lineage-committed Th-1/Th-17 cells as evidenced by a decrease in the production of IFN-γ and IL-17 by CD4^+^ T cells, which is in agreement with the literature [63].

In conclusion, we found that ADSC inhibited IL-17 production and expanded Treg *in vivo* thereby generating improved allograft survival in a skin transplant model. Prolonged transplant survival was associated with higher expression of IL-6, IL-10 and IFN-γ. As ADSC are plentiful in patient adipose tissue, the isolation and deployment of an ADSC therapy is an attractive methodology for the clinical prolongation of allograft survival.
